# Human circulating bacteria and dysbiosis in non-infectious diseases

**DOI:** 10.3389/fcimb.2022.932702

**Published:** 2022-08-24

**Authors:** Mohsan Ullah Goraya, Rui Li, Abdul Mannan, Liming Gu, Huixiong Deng, Gefei Wang

**Affiliations:** ^1^ Guangdong Provincial Key Laboratory of Infectious Diseases and Molecular Immunopathology, Shantou University Medical College, Shantou, China; ^2^ School of Biomedical Sciences and Pharmacy, College of Health, Medicine and Wellbeing, University of Newcastle, Callaghan, NSW, Australia

**Keywords:** blood microbiota, transient bacteremia, blood bacteria and dysbiosis, 16S rDNA, diabetes, cardiovascular diseases

## Abstract

Blood microorganisms were once thought to indicate infection. Blood in healthy people appears to be devoid of growing bacteria; nonetheless, intracellular dormant forms of bacteria have been reported previously. With breakthroughs in sequencing and bioinformatics, the presence of bacterial DNA in healthy human blood initiated the controversy of human blood microbiota (HBM). Recently, bacteria-specific DNA and culturable bacteria were found in healthy human blood. Researchers wanted to study the phenomena of a “healthy blood microbiota” by providing a thorough description of bacterially produced nucleic acids using many complementing molecular and traditional microbiological approaches. Because blood is a relatively limited and particular environment, culturability and plate count issues can be overcome using enhanced cultured procedures. However, more evidence is required to confirm that healthy human blood contains normal microbiota. Cavities, mouth and intestinal microbiota, trauma, surgery, and animal/insect bites can introduce bacteria into human blood. All these factors strengthen the concept of transient blood bacteria too. The presence of blood bacteria may be caused by temporary immunological clearance and absorption by dendritic or M cells. This review provides an extensive and comprehensive analysis that suggests that healthy blood bacteria may not be typical microbiota but transient circulatory microorganisms. In this study, we look at how contaminants (*Escherichia*, *Shigella*, *Pseudomonads*, etc.) from the skin, laboratory environments, and reagents can affect the interpretation of blood-derived microbial information and the relationship between the circulating bacteria and non-communicable diseases. Circulating transient bacteria may play a role in the pathogenesis of non-infectious diseases such as diabetes and CVD. Contamination-free hematological studies can aid in understanding the disease mechanisms, therapy, and biomarkers.

## Introduction

Over the last two decades, studies on the human microbiome have been getting huge consideration. Enormous numbers (10–100 trillion) of microorganisms colonize significant parts of the human body, such as the skin, oral cavity, gastrointestinal tract (GIT), respiratory tract, and urogenital tract. Microbiome research received much attention due to the long-lasting effects on significant health issues, including metabolism, depression, blood pressure, brain health, and chronic infectious diseases. Through the evolutionary process, many microbial species have successfully adapted to the normal microbiome of humans. Most of these microbes could not be cultured in *in vitro* environments. Inside the human body, these microbes multiply and participate in essential biological processes and set the bases for several communicable and non-communicable human diseases. At the same time, the colonization of microbes in the body compartments continuously in contact with external microorganisms (such as mouth, respiratory tract, GIT, skin, urethra, and vagina) is well studied and widely accepted (Markova, 2017).

The human gut harbors the most complex human microbiota niche, most of which belong to the Gram-positive *Firmicutes* and *Actinobacteria*, while among the Gram-negative phyla *Bacteroidetes* and *Proteobacteria, Fusobacteria* are most abundant and *Euryarchaeota* are found in minor quantities. However, it varies among individuals due to certain factors such as diet, age, geographical location, use of antibiotics, and polluted drinking water (Arumugam et al., 2011; Segata et al., 2012; Brown et al., 2013). The gut microbiota, which might be changeable, performs different functions in the human body, including digestion and metabolite production. Human microbiota helps establish the early immune system, modulation, and protection against pathogens (Clemente et al., 2012; Brown et al., 2013). Similarly, oral microbiota comprises more than 300 genera from different parts of the buccal cavity (Zhou et al., 2013). It is expected that disease conditions such as periodontitis and caries construct complex microbiota consisting of Gram-negative anaerobic bacteria, including *Porphyromonas gingivalis*, *Treponema denticola*, *Prevotella intermedia*, *Tannerella forsythia*, and *Agregatibacter actinomycetemcomitans* coexisting with Gram-positive anaerobic bacteria (Mason et al., 2013; Jiang et al., 2014). The skin microbiota is composed of over 100 phylotypes, most of which are non-pathogenic. According to geographical location, environmental conditions, and vocations, the colonization of skin microbiota might differ significantly among individuals. Among the skin microbiota, *Actinobacteria* are the most prevalent phylum of bacteria. Likewise, vaginal microbiota comprises more than 200 phyla and is dominated by *Firmicutes*, *Bacteroidetes*, *Actinobacteria*, and *Fusobacteria* (Romero et al., 2014), while there is less information available about the microbiota of circulatory fluids of confined human body compartments, such as spinal fluid, blood, and other minor secretions of the human body. Recently, it is suggested that blood uptakes healthy bacteria and their metabolites while it circulates throughout the human body.

Microbial populations (including pathogens) in circulatory fluids of confined compartments (classically considered as sterile), including the spinal fluid and blood, are a relatively new and barely studied concept (Ghose et al., 2019; Lathe and St Clair, 2020; Kang et al., 2021; Liao et al., 2021). In the past, blood microbes were unanimously considered an indication of infection. Conversely, with advances in sequencing and bioinformatics tools, Schierwagen and coworkers analyzed the portal venous blood microbiota *via* the liver, central venous blood, and peripheral blood (PB) in seven patients with decompensated liver cirrhosis. Buffy coat (BC) 16S rRNA gene sequencing discovered 65 genera across four phyla (predominantly Proteobacteria) (Schierwagen et al., 2019). However, postscript response emphasized that essential controls are required for blood microbiota research (Schierwagen et al., 2020). Human blood runs throughout the body without interacting with the external environment, except for the uptake of nutrients, microbial metabolites, and microbes from the epithelial cells of GIT and other body tissues. Previously, studies showed traces of bacterial DNA in healthy individuals (Nikkari et al., 2001; Païssé et al., 2016), but due to the concept of sterile blood, and high chances of contamination, blood microbiota has been criticized widely. However, the blood microbiota in various domesticated animals and birds has been observed and reported (Mandal et al., 2016; Vientós-Plotts et al., 2017; Scarsella et al., 2020). The evidence of foreign cells in healthy human blood was reported in the late 1960s, and the presence of a metabolically active form of mycoplasma or bacterial L-forms in the blood of clinically healthy humans was detected (Tedeschi et al., 1969). Subsequently, in 1977, researchers found bacterial traces in the blood of healthy subjects (Domingue and Schlegel, 1977). However, the concept was criticized by some researchers. For instance, Mitchell et al. (2016) argue that pleomorphic bacterial-like structures were erythrocyte-derived micro-particles and 16S rDNA sequences were the laboratory contaminants (Mitchell et al., 2016). In another corresponding study, Martel et al. (2017) suggested that the observed bacteria-like structures in human blood are the artifacts of non-living membrane vesicles and aggregates of blood-derived proteins (Martel et al., 2017).

In the 21st century, findings based on new technologies further strengthen the notion that bacterial DNA is present in healthy individuals. In 2001, Nikkari and colleagues found bacterial DNA in the blood of healthy individuals by qPCR, consisting of rRNA-specific fluorescent probes and primers to target the conserved regions of 16S ribosomal DNA of bacteria (Nikkari et al., 2001). Sterile water was filled in vacutainer tubes used as a negative control. It was unclear if the bacterial DNA in blood came from skin, blood, or both. They also suggested to avoid PCR reagent’s background bacterial DNA contamination and to evaluate the most abundant DNA present in PCR reagents. The fractionation of whole blood of healthy individuals identified that of all bacterial DNA, 93.74%, 6.23%, and 0.03% were found in the BC, red blood cells, and plasma (Païssé et al., 2016), respectively. *Proteobacteria* DNA was the most abundant (more than 80%), including *Actinobacteria*, *Firmicutes*, and *Bacteroidetes*. Using 16S rRNA (rDNA)-specific primers, Moriyama et al. (2008) found a diversified clone of the bacterial population, including *Aquabacterium, Stenotrophomonas*, *Budvicia, Serratia, Bacillus*, and *Flavobacteria*, while no traces of GIT endogenous (*bacteroides*, *clostridium*, and *lactobacillus*) cluster were found (Moriyama et al., 2008). These shreds of evidence initiated the concept of bacterial DNA existence in healthy human instead of conventional thoughts of considering blood as a sterile fluid. Although the presence of blood microbiota requires visual and viable confirmation, studies providing the evidence of bacterial genetic material in healthy human blood and its role in non-communicable diseases are increasing (Païssé et al., 2016; Panaiotov et al., 2018; Whittle et al., 2019; Velmurugan et al., 2020). However, it is unclear whether the bacteria found are the blood microbiota or transient bacteremia as blood cells regularly uptake the bacteria from gut, mucosa, or local infections. Application of robust, innovative technologies, including 16S rRNA sequencing and metagenomics, can improve the blood bacteria detection in detail. Parallel to this, considerable attention must be paid to experimental controls to minimize the effect of unknown factors when studying the microbiome, as the samples are prone to contamination from environmental sources.

## How bacteria get into sterile blood

The blood is considered sterile and lacks culturable bacteria. It serves as the best-quality growth medium for *in vitro* culture of bacteria, since as few as 1–10 ml^−1^ bacterial cells in blood could be life-threatening during any infection (Bennett et al., 2019). However, few recent studies questioned the sterility of blood in healthy individuals, as it does not imply the lack of latent or non-cultural organisms. In a study, 71% of blood samples obtained from sick individuals and 7% from allegedly healthy people were reported to have a new bacteriological niche (Domingue and Schlegel, 1977). In 2001, samples from healthy individuals were shown to contain 16S bacterial ribosomal DNA following the detection of corynebacteria-like microorganisms in RBC hemocultures (Nikkari et al., 2001). These studies debated whether blood-borne bacteria are valid biological niches or just transitory blood-borne inhabitants. Some experts believe that bacteria in the blood result from translocation from other bodily locations, notably the gastrointestinal system.

**Figure 1:**
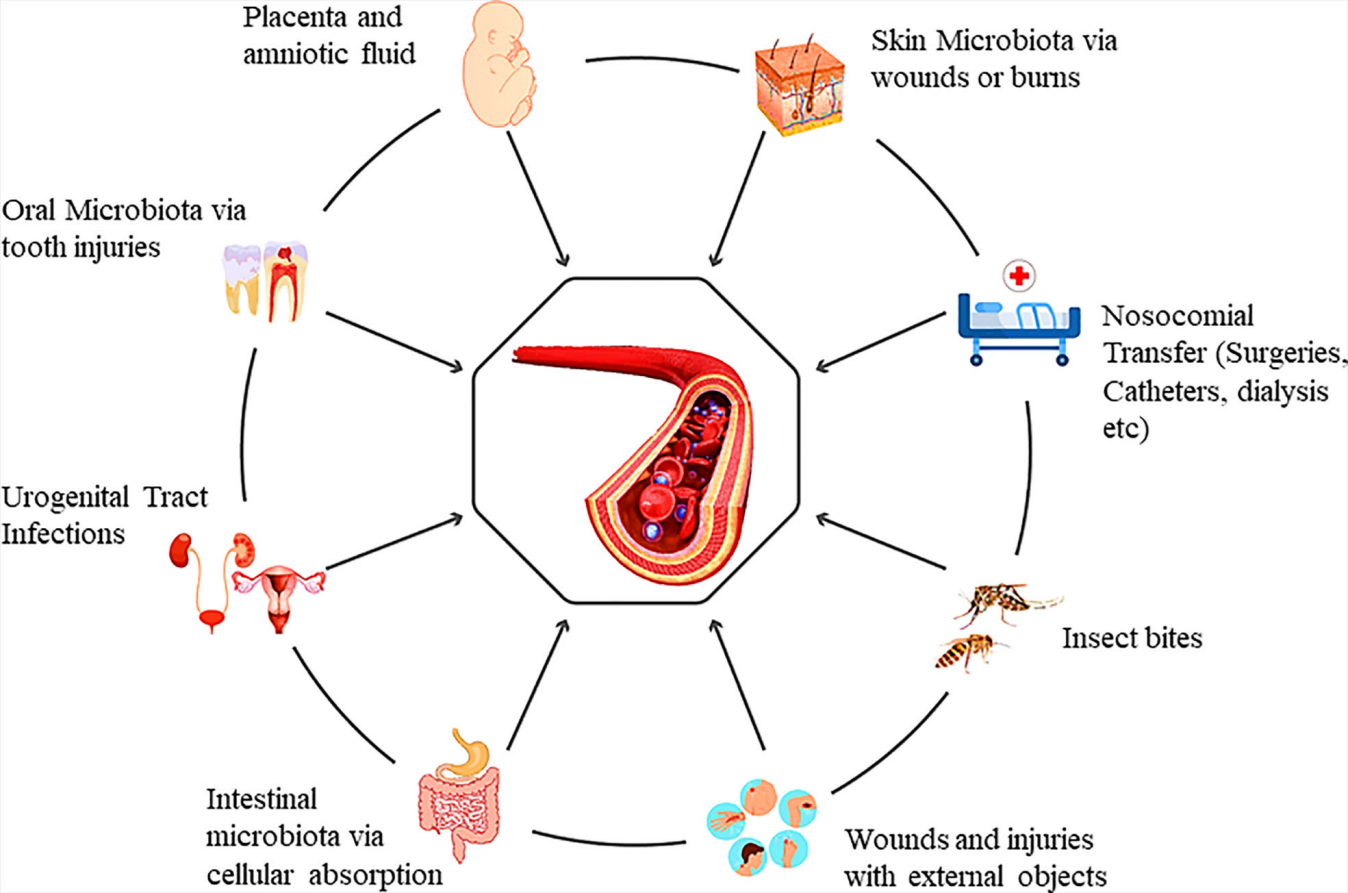
Genesis of the blood microbiota and the multiple possible entry portals.

Primarily, the presence of bacteria in the blood could be the outcome of microbial translocation from the microbiota enriched locations of the human body, or they can enter during clinical procedures (physical or surgical). Besides these, humans can also acquire a variety of microbes from the environment directly into the human blood (physical injuries, insect bites, scratches, or animal bites and brushing of teeth) (**Figure 1**). The first-ever entry of the microbes into infants’ blood can occur during gestation. There is evidence that the fetal surroundings, such as amniotic fluid, placenta, fetal membranes, and umbilical cord blood, may have microorganisms (Funkhouser and Bordenstein, 2013). Blood from the maternal side cannot be mixed with fetal blood during gestation. Still, there is evidence that maternal bacteria can be found in fetal blood during pregnancy. The uterus has long been considered sterile, while bacteria have been cultured from the umbilical cord blood samples (Jiménez et al., 2005). This study also favors the concept of healthy circulatory transient bacteria transferred from mother to fetus. In another study, to check the transfer of maternal bacteria to fetus, pregnant mice were fed with genetically tagged *E. fecium* strain from women’s breast milk. PCR validated the tagged strain in pups’ meconium (Jiménez et al., 2008). In contrast, some believe that the fetal microbiome is acquired through the uterus and enriched after birth (Romano-Keeler and Weitkamp, 2015).

The oral microbiota can enter the blood when the tight junctions of cells are compromised (Iwai, 2009) or the gums are damaged during the brushing of teeth. Skin bacteria can also escape into the blood in case of any damage to the skin barrier (Cogen et al., 2008). Comparing human microbiome project (HMP) data and healthy human blood microbiota mostly shares microbial DNA of skin and oral microbiota (Whittle et al., 2019). However, previous studies claim that DNA analysis showed significant variation from the gut microbiota. We believe that it can be due to the diffusion of bacteria into blood circulation as an unusual incidence, which may frequently occur in healthy people. Usually, it is considered that bacteria can enter blood circulation only when the epithelial barriers are compromised. However, even if the intestinal membranes are intact, bacteria can enter the circulatory system through different intestinal and circulatory cells (Wiest et al., 2014). Dendritic cells, for instance, can uptake microbial products *via* crossing processes between epithelial cells without affecting tight junction function (Niess et al., 2005). These are the primary routes that challenge blood sterility. Intestinal mucus-secreting goblet cells and mucosal lymph cells (specialized epithelial cells of the mucosa-associated lymphoid tissues) lying over Peyer’s patches also act as the microbial carrier from the intestine to blood circulation (Castillo et al., 2019). To date, we have mere information regarding the transfer of bacteria to blood *via* general injuries, surgical and non-surgical procedures at hospitals, and animal or insect bites. Extensive studies are required to unveil the acquisition of healthy blood microflora from different possible sources. Major pilot studies from different countries across the continents can solve this myth to some extent. Recently, a pilot study reported the presence of blood microbiome in a generally healthy population recruited under the MARK-AGE project of European countries (D’aquila et al., 2021). Additionally, researchers have to investigate the escape of lung microbiota from lung tissues to the blood. As blood continues to circulate, it is likely to contain transitory bacteria or bacterial DNA.

## Human presumptive blood microbiota profile

**Figure 2:**
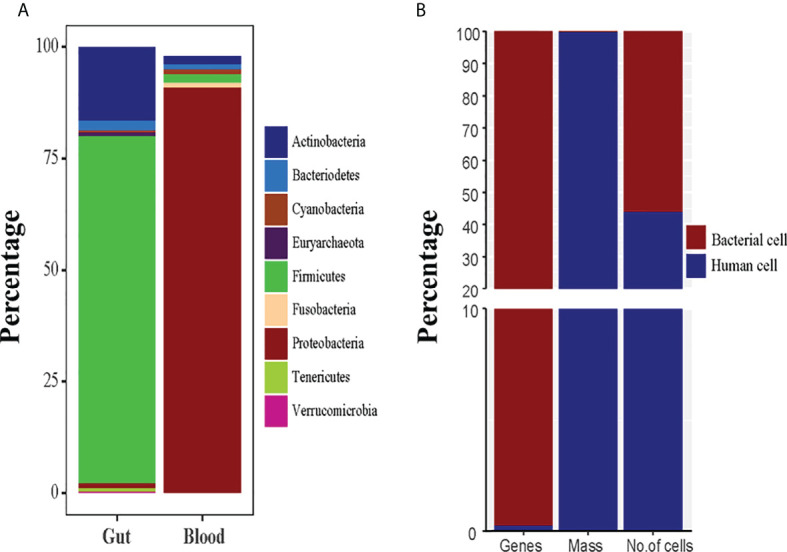
**(A)** The normal healthy microbiota of the human gut and blood are compared in terms of microbial diversity. **(B)** The human body system and the human microbiota are contrasted in terms of weight, cells, and genes.

Knowing if and how bacteria may survive in the blood is critical because blood is a hostile environment for germs due to bacteriostatic and bactericidal components (Markova, 2017). It is considered that microbiota can be taken up from the intestine while the composition of healthy blood bacteria is different from the intestinal microbiota. Unlike the intestinal microbiota, where *Firmicutes* and *Bacteroidetes* are the most common bacterial phyla, the reported blood microbiota is dominated by the *Proteobacteria*, followed by *Actinobacteria*, *Firmicutes*, and *Bacteroidetes* (Velmurugan et al., 2020). When it comes to blood microbiota compositions, there are some commonalities among them: *Proteobacteria* predominates (with relative abundance values typically ranging from 85% to 90%), while *Firmicutes*, *Actinobacteria*, and *Bacteroidetes* show up in much smaller numbers (**Figure 2A**) (Amar et al., 2013; Dinakaran et al., 2014; Qiu et al., 2019). There are reservations that blood samples may be contaminated by the laboratory environment, DNA extraction kits, or equipment. However, the studies published to date have similar results at the phyla level, suggesting that a core blood microbiota profile may exist and is independent of the research environment or analysis approach. However, several considerations need to be taken into account, including transient bacteremia, immune clearance *via* macrophages, and contamination from skin, equipment, or reagents while studying blood microbiota. Early studies only detected L-form bacteria (Tedeschi et al., 1969), and growing phases of bacteria and pleomorphic antibiotics-susceptible bacteria (Domingue and Schlegel, 1977; Mclaughlin et al., 2002) in the blood with old-fashioned technologies. The new and innovative molecular techniques are being used to discover non-culturable bacteria from human microbiota, showing the wide range of individual differences among the microorganisms. The revised bacteria-human cell ratio (B/H= 1.3), indicate that bacterial cells are slightly more than the human cells (Sender et al., 2016). Though microbial cells are more numerous and smaller in size than human cells, they account for only 0.3% of human body weight and over 99% genes (**Figure 2B**) present in human body (Qin et al., 2010; Sender et al., 2016).

Recent studies have comprehensively characterized bacterial diversity among healthy human blood. In a culture-based study by Damgaard et al. (2015), *Propionibacterium acnes* were the most prominent taxa found in the blood of 62% of healthy individuals, followed by *Staphylococcus epidermis* and *Bacilli* and *Micrococcus* species (Damgaard et al., 2015), which might be due to contamination of skin microbiota as these bacteria are common skin microbiota. However, *Bacillus, Flavobacteria, Stenotrophomas*, and *Serratia* were the most prevalent taxa found in an earlier study of two healthy individuals’ blood (Moriyama et al., 2008). A study was designed to characterize the bacterial diversity in blood fraction (RBCs, BC, and plasma) of 32 healthy individuals at the French national blood collection center (Etablissement Français du sang). The blood bacteria were characterized by 16S rDNA qPCR and 16S metagenomics. The majority of the blood bacterial DNA (93.74%) was found in the BC as compared to RBCs (6.23%) and plasma (0.03%). Blood fractions include bacterial DNA primarily from the *Proteobacteria* phylum (more than 80%) and *Actinobacteria, Firmicutes*, and *Bacteroidetes*. At a higher taxonomic level, the bacterial profiles of the different blood fractions differ dramatically. *Fusobacteria* and *Flavobacteria* were abundant in RBCs, while *Actinobacteria, Bacilli*, and *Clostridia* members were more prevalent in plasma and erythrocyte fractions than BC (Païssé et al., 2016).

The presence of bacterial DNA can be the outcome of immune or intestinal cell-related bacteria or free bacterial DNA due to transient event of immune clearance. The *Sphingobacteria* class is predominantly found in BC and plasma. Similarly, a study was designed to analyze the blood bacteria in elderly subjects; interestingly, the results were in line with previous studies. Most of the bacterial DNA found in blood samples of elderly subjects belonged to the phyla *Proteobacteria* and *Actinobacteria*, while *Firmicutes* and *Bacteroidetes* were underrepresented and were not found in all subjects (Gargari et al., 2021). Bacterial DNA-based results of all these studies are almost similar, which urges scientists to study more to establish the details of blood microbiota. However, bacterial DNA contamination from extraction kits and reagents has been found in bacteriology laboratories and often comprises *Bacillus, Flavobacteria, Fusobacteria, Propionibacterium*, and *Serratia* (Glassing et al., 2016). These studies have tested the reagents and designed particular protocols to minimize contamination. Blood microbiota affects host physiology and health, regardless of origin (skin, stomach, oral cavity, placenta, or lung) or form of bacterial DNA (free DNA, free bacterial cells, or bacteria internalized in blood cells). The blood bacterial populations reported in several studies are stable regardless of age or gender, with minor alterations due to past disease, surgery, and non-infectious and metabolic diseases. Non-communicable and metabolic disorders have altered the blood microbiome, according to studies (Qiu et al., 2019; Shah et al., 2019). These findings may support the presence of bacterial DNA in healthy humans’ blood and lack the evidence for live bacteria due to culture limitations. Still, its source must be determined (transient bacteria, accidental uptake, or contaminations). Blood microbiota is only significant and scientifically intriguing if it represents an undisturbed condition, not background contamination, WBC- or RBC-related bacteria, or immune clearance.

## Is blood microbiota consistent with transient bacteremia

The study of blood microbiota is even more challenging because of the low concentration of bacterial DNA, higher chances of contamination during phlebotomy, and background contamination. Therefore it requires highly sterile laboratory equipment and advanced molecular techniques. The use of microbial blood culture, quantitative PCR, next-generation 16S rRNA gene sequencing (Moriyama et al., 2008; Amar et al., 2013; Païssé et al., 2016), shotgun metagenome sequencing (Kowarsky et al., 2017), and 16 rRNA gene targeted sequencing Illumina MiSeq (Panaiotov et al., 2018; Qiu et al., 2019) has provided the supporting evidence of the existence of diverse bacterial population in healthy human blood. There are considerable differences between the blood bacteria found in healthy and diseased individuals although samples were being processed equally. Certain diseases appear to be linked to an organism’s morphology (for example, coccus versus bacillus). Pleomorphic forms of different pathogenic bacteria such as coccus or bacilli are present in diseased individuals, while no such variation is found in healthy individuals. Various studies of healthy blood bacteria in different laboratories have reported a similar composition of microbial phyla and slight variations at the class level. Collectively, these results minimize the possibilities of contamination. In a separate study, blood and neutrophil-associated microbiota were characterized by Qiurong Li and colleagues in patients with severe acute pancreatitis (SAP). *Bacteroidetes* and *Firmicutes* were more prevalent in patients’ blood and neutrophil-associated microbiota than in healthy controls, though *Actinobacteria* were less prevalent. While there was no statistically significant difference in bacterial composition between patient subgroups, these findings suggest that changes in the blood bacteria may not be associated with the presence or absence of infectious complications in SAP (Li et al., 2018).

In clinical microbiology studies, molecular techniques are being used extensively to detect blood bacteria in diseased or healthy individuals. Culture- and molecular method-based studies found that the BC samples of patients with liver cirrhosis were dominated by proteobacteria, consistent with the previously reported blood microbiota of healthy individuals (Païssé et al., 2016) and patients with liver fibrosis (Lelouvier et al., 2016). Different comparison studies of diseases and healthy individuals reported the same blood microbiota of healthy individuals (**Table 1**). In a commentary, Hornung et al. (2020) proposed that those culturable strains of *Staphylococcus* and *Acinetobacter* are known contaminants of skin and water, respectively (Hornung et al., 2020). In response, Schierwagen and colleagues explained that the bacterial genera listed by Hornung et al. (2020) as possible contaminants were not the result of any contamination in their study. These bacteria were absent or significantly lower in negative controls than in blood samples (Schierwagen et al., 2020).

**Table 1:** Summary of different human blood microbiota studies conducted on healthy or diseased individuals.

Study Population	Health Indications	Sample	Methodology	Findings	Ref.
**Healthy individuals**					
A total of 100 men and women were chosen at random.	Only healthy individuals were selected	RBCs	Radioactive uptake of nucleosides and amino acids by RBCs	Finding of L-phase bacterial forms in the healthy blood	(Tedeschi et al., 1969)
4 healthy individuals	Healthy individuals only	Whole blood	qPCR and rRNA gene-specific fluorescent probes targeting the conserved region of 16S rDNA	Bacteria from five divisions and seven distinct phylogenetic groups detected in the blood	(Nikkari et al., 2001)
25 healthy individuals.	Healthy individuals only	Whole blood	Characterization by 16S rRNA and *gyrB* genes and detected by dark-field microscopy and fluorescent *in situ* hybridization (FISH)	Pleomorphic antibiotic susceptible bacteria existing in healthy blood with limited growth (possibly *Pseudomonas*)	(Mclaughlin et al., 2002)
2 healthy individuals	Healthy individuals only	Whole blood	16S rRNA PCR and Sanger sequencing	*Aquabacterium, Budvicia*, *Stenotrophomonas, Serratia*, *Bacillus*, and *Flavobacteria* identified only in clones	(Moriyama et al., 2008)
60 self-reported healthy individuals’ ≥49 years.	Almost 64% were positive for bacterial growth	Blood plasma and RBC suspension	Blood suspensions incubated on TSA or blue lactose plates, and 16S rRNA gene colony PCR used to identify bacteria	Bacterial growth observed in 35% of RBC fractions and 53% of plasma fractions. *Staphylococci*, *Propionibacterium*, *Micrococcus*, and *Bacillus* most frequently found	(Damgaard et al., 2015)
30 healthy blood donors (18 to 53 years old).	Healthy blood donors	Blood fractions (buffy coat, plasma, and RBCs)	16S rRNA gene qPCR and 16S targeted metagenomic sequencing (Illumina MiSeq)	Buffy coat, erythrocytes, and plasma were positive for bacterial DNA. Most prevalent bacterial DNA belong to *Proteobacteria* and *Actinobacteria (Firmicutes* and *Bacteroidetes* also found)	(Païssé et al., 2016)
28 blood samples from healthy individuals	Healthy individuals only	Whole blood, positive for bacterial cultures	16S rRNA genes and ITS2 targeted sequencing on Illumina MiSeq and TEM	Cultural and molecular characterization of healthy blood microbiota (*Proteobacteria* and *Basidiomycota* were prominent)	(Panaiotov et al., 2018)
**Diabetic vs. healthy individuals**					
119 diabetic and 480 non-diabetic patients	Diabetic and non-diabetic individuals	Whole blood	Aerobic and anaerobic blood cultures	Diabetes patients have higher Klebsiella and Staphylococci	(Leibovici et al., 1991)
3,280 people without diabetes and obesity at baseline (9 years observation)	Non-diabetic patients with bacterial DNA in their blood	DNA extracted from leukocytes (peripheral blood)	16S rDNA quantitative PCR and pooled pyrosequencing	Regardless of any risk factors, individuals with high 16S rDNA levels developed diabetes. High prevalence of *Ralstonia* spp. in individuals who developed diabetes	(Amar et al., 2011)
50 diabetic and 50 non-diabetic individuals	Type 2 diabetes patients and control individuals	Circulating RNA isolated from blood plasma	Measurement of 16S rDNA and genus-specific 16S rDNA by qRT-PCR	High 16S bacterial rRNA content in diabetes patients; *Clostridium coccoides* and the *Atopobidum* cluster were particularly abundant	(Sato et al., 2014)
50 diabetic and 100 non-diabetic Individuals	Diabetic and healthy individuals selected by pre-diagnostic analysis	Circulating DNA isolated from blood plasma	16S rRNA amplicon sequencing by Illumina MiSeq	*Bacteroides* spp. showed an inverse correlation and *Sediminibacterium* spp. showed a positive correlation with diabetes	(Qiu et al., 2019)
30 healthy people, 30 type 2 diabetes, and 30 pre-diabetic people	Pre-diabetic and healthy individuals	Buffy coat	Real-time PCR using genus-specific 16s rRNA primers	*Akkermansia* and *Faecalibacterium* were higher in healthy individuals compared to pre-diabetic and type 2 diabetes	(Ghaemi et al., 2021)
1,285 RASIG individuals under MARK-AGE an EU project (2008-2012).	Seropositive individuals for HCV, HIV, cancer	DNA extracted from whole blood	Quantification of 16S rRNA by real-time qPCR	High level of bacterial DNA was associated with higher level of insulin and glucose	(D’aquila et al., 2021)
**Cardiovascular vs. healthy individuals**					
1,312 incident coronary heart disease patients and 727 incident stroke patients	Patients with cardiovascular problems	DNA extracted from peripheral blood leukocytes	Analysis of Atherosclerosis Risk Communities study (ARIC) results over the period 1987–2017	Inpatient and outpatient infections are associated with CVD risk	(Cowan et al., 2018)
3,936 people without diabetes or obesity at baseline	Bacterial DNA in blood of individuals not presenting CVD.	DNA extracted from leukocytes (peripheral blood)	Measurement of *Eubacteria* and *Proteobacteria* 16S rDNA by qPCR	There was a positive correlation of Proteobacteria, and inverse correlation of Eubacteria, with cardiovascular events	(Amar et al., 2013)
31 CVD patients and 10 healthy controls	CVD and healthy individuals with no history of antibiotics (30 days)	DNA extracted from whole blood	Amplicon sequencing of 16S rDNA (Ion Torrent PGM)	Increase in Pseudomonadaceae and decrease in Gammaproteobacteria, Bacillales, and Staphylococcaceae in CVD patients	(Rajendhran et al., 2013)
80 CVD patients and 40 healthy blood donors	Healthy individuals have bacterial DNA in their plasma	Circulating DNA isolated from blood plasma	Measurement of total 16S rDNA and β-globin gene concentrations by qRT-PCR. Shotgun sequencing of DNA and amplicon sequencing of 16S rDNA (Ion Torrent PGM)	The 16S rRNA/β-globin gene ratio was higher in CVD patients than in controls. *Actinobacteria* and Bacteriophages were dominant in CVD patients whereas *Proteobacteria* and eukaryotic viruses were dominant in controls	(Dinakaran et al., 2014)
**Miscellaneous**					
23 healthy individuals and 62 patients with sepsis	All were positive for bacterial DNA	Whole blood	16S rRNA gene targeted metagenomic NGS	Healthy samples presented higher diversity than sepsis patients. *Proteobacteria* were lower in healthy individuals, while *Actinobacteria* decreased in sepsis patients	(Gosiewski et al., 2017)
9 cirrhotic and nine healthy individuals (≥60 years)	Bacteria found in two healthy individuals	Blood (plasma)	16SrRNA target gene qPCR	Bacterial biodiversity and amount of bacterial DNA increased in cirrhotic patients	(Traykova et al., 2017)
50 patients with severe acute pancreatitis and 12 healthy individuals	Bacterial DNA present in all healthy participants	DNA from whole blood and neutrophils	16S rDNA gene qPCR and targeted metagenomic sequencing using Ion Torrent.	16S rDNA gene copies were higher in patients. Healthy phyla include *Proteobacteria*, *Actinobacteria, Firmicutes* and *Bacteroidetes. Bacteroidetes* were high and *Actinobacteria* lower in patients	(Li et al., 2018)
192 individuals (48 with schizophrenia, 47 with lateral sclerosis, 48 with bipolar disorder and 49 healthy).	Bacterial DNA present healthy individual’s blood	Whole blood	High-quality unmapped RNA sequencing	The most prevalent phyla among the groups were *Proteobacteria, Firmicutes*, and *Cyanobacteria*, and schizophrenia patients have high microbial diversity.	(Olde Loohuis et al., 2018)
Healthy and asthma patients (five each, all women)	Bacterial transcripts in blood of all healthy individuals	Plasma fractions	16S rRNA gene sequencing. *De novo* assembly of unmapped mRNA reads, and culturing	Most abundant phyla were *Proteobacteria*, *Actinobacteria*, *Firmicutes*, and *Bacteroidetes*	(Whittle et al., 2019)

In many cases, relevant organisms are found during intracellular or transient episodes of bacteremia, which is difficult to explain through contamination. Though the skin or laboratory contamination will remain a serious concern, many studies have proposed the blood microbiota with the help of advanced molecular techniques in blood and serum, all of which use proper and cautious controls. The same methods used to detect the bacteria in blood cultures during sepsis is also considered the standard diagnostic tool for bloodstream infections (Muñoz et al., 2008; Varani et al., 2009); this supports the fact that the careful sample collection and appropriate negative controls can be helpful to study the healthy blood microbiota.

## Healthy blood bacteria dysbiosis in chronic diseases

Since blood microbiota does not induce complications like inflammation and sepsis, it may play an essential role in normal physiology and immunity. Previously, the majority of non-culturable forms of bacteria found in wounds and certain diseases such as cystic fibrosis or tuberculosis are “normally culturable” now (Potgieter et al., 2015). Dormancy is well-known in microbiological research and is critical when studying microbial ecology. Blood microbiota may not cause infections or other complications because it is dormant (Potgieter et al., 2015). However, it might be related to dysbiosis in some conditions. The term “dysbiosis” refers to a change in symbiotic or commensal microbial communities (Petersen and Round, 2014; Levy et al., 2017). Yet, it is unclear whether dysbiosis is a cause of a disease or simply a reflection of it (Bäckhed et al., 2012; Yoo et al., 2020).

To date, dysbiosis of intestinal microbiota in diabetes (Xie et al., 2021), CVD (Zhu et al., 2020), asthma (Lu and Zou, 2020), and complex inflammatory diseases, including Alzheimer’s disease (Shabbir et al., 2021; Zhang et al., 2021) and Parkinson’s disease (Peng et al., 2021), has been discussed extensively. Non-communicable and metabolic diseases that impact bacterial translocation and dysbiosis in the blood are listed in **Table 1**. In contrast, little research has been conducted to uncover the role of blood microbiota dysbiosis in metabolic or cognitive disorders. In most cases, blood is protected from intestinal microbes and microbial products by gut immunological barriers and intestinal cells. The reticuloendothelial system attempts to clear microbes, microbial metabolites, or toxins passed through the gut barrier into the bloodstream. If this is unsuccessful, dysbiosis of blood bacteria may lead to chronic inflammation, which leads to metabolic disorders including diabetes, pancreatitis, liver cirrhosis, and CVD. Patients with Alzheimer’s disease have periodontitis-causing bacterium *P. gingivalis* in their brain tissue, indicating that the bacteria had moved from the oral cavity to the brain through the bloodstream (Dominy et al., 2019). A number of studies have been conducted to investigate the link between blood microbial dysbiosis and chronic illnesses. Blood bacteria and bacterial lipopolysaccharides (LPS) are associated with diabetes. Patients with type 2 diabetes mellitus have significantly higher levels of the LPS-binding protein (LBP), and LBP was positively correlated with the glycated hemoglobin, body mass index, and inflammatory markers of study participants (Donath and Shoelson, 2011).

In a longitudinal study of 3,280 non-diabetic individuals, higher baseline levels of blood bacterial 16S rDNA were found as an independent risk factor for the onset of type 2 diabetes. However, the same study reported no significant difference between the HBM of healthy and diabetic individuals (Amar et al., 2011). The pyrosequencing analysis revealed a higher abundance of *Proteobacteria* in both type 2 diabetes mellitus and non-diabetic individuals, with diabatic individuals having a higher abundance of *Ralstonia* spp. (Amar et al., 2011), while subsequent studies have found detailed variations at the genus level in gut microbiota and blood plasma and cellular levels. Patients with pre-diabetes or type 2 diabetes have fewer *Faecalibacterium*, *Akkermansia*, and *Bifidobacterium* bacteria in their leukocytes than healthy people, according to an epidemiological study on Iranian diabetes patients. The bacterial loads of *E. coli* and *Bacteroides fragilis* were higher in pre-diabetic individuals than healthy individuals, while type 2 diabetes groups had higher *Lactobacillus*, *E. coli*, and *Bacteroides fragilis* (Ghaemi et al., 2021). However, according to a case study in the Chinese population, no differences were reported in the baseline microbiota diversity of diabetes and control participants. The same study reported significant variations in diversity at the genus level. For example, the relative abundance of *Aquabacterium, Xanthomonas*, and *Pseudonocardia* was low and that of *Actinotalea, Alishewanella, Sediminibacterium*, and *Pseudoclavibacter* was highly prevalent in T2DM patients compared to healthy individuals. The genus *Bacteroides* was inversely correlated and *Sediminibacterium* was positively correlated with the risk of diabetes (Qiu et al., 2019). IM bacteria have been found in 28% of diabetes patients in a study, but healthy participants had only 4% of the IM-derived bacterial load.

Similarly, individuals with type 2 diabetes had higher blood plasma, and gut microbiota levels of Gram-positive bacteria (specifically *Clostridium coccoides* and the *Atopobium cluster*) were significantly higher in type 2 diabetes patients than in healthy controls (Sato et al., 2014). Recently, a European study of 1,285 individuals showed that higher levels of blood bacterial DNA were linked to higher blood glucose and insulin levels in a randomly selected age-stratified general population (D’aquila et al., 2021) (**Table 1**). These studies suggest a causal link between the onset of diabetes and HBM dysbiosis. The findings suggest that dysbiosis of blood bacteria can be used as a biomarker for the early diagnosis and control of diabetes.

In an epidemiological study, gut bacterial DNA was examined in blood using 16S rRNA and IM Microbial qPCR microarray;, 90% of patients with liver cirrhosis had higher gut microbiota diversity than healthy individuals. The diseased cohort’s blood had a higher total bacterial DNA concentration than healthy controls (Traykova et al., 2017). Additionally, researchers used the LDA effect size (LEfSe) method to examine the circulating blood bacteria of patients with liver cirrhosis. Enterobacteriaceae abundance in PB patients with liver cirrhosis was higher than healthy individuals. At the same time, the levels of *Akkermansia*, *Rikenellaceae*, and *Erysipelotrichales* were significantly higher in the PB of healthy individuals compared to cirrhotics (Kajihara et al., 2019). An increase in *Bacteroidetes* and *Firmicutes* was observed, while an overall decrease in *Actinobacteria* was observed in pancreatitis patients compared to the healthy human cohort. *Bacteroidia* and *Clostridia* numbers increased in the diseased group while *Actinobacteria*, *Flavobacteria*, and *Bacilli* numbers decreased. However, no significant composition differences were found between patient subgroups (Li et al., 2018). This suggests that changes in the microbiota are not linked to the presence or absence of infectious complications in SAP, and discrepancies in dominant taxa in patients with pancreatitis have a dysbiotic blood microbiota. Researchers have also hypothesized that blood microbiota could risk developing nonalcoholic fatty liver disease (NAFLD) (Lelouvier et al., 2016; Yun et al., 2019). It was found that patients with liver fibrosis (LF) had higher levels of the 16S rDNA gene in their blood than healthy individuals. Analysis of the LEfSe algorithm showed that the patients with LF had lower proportions of *Actinobacteria* than healthy controls, whereas *Proteobacteria* was higher in the same group (Lelouvier et al., 2016).

Blood bacterial dysbiosis may cause cardiovascular diseases. Microbes’ role in CVD was previously limited to pathogen-infected complications like rheumatic carditis, pericarditis, myocarditis, and endocarditis. A recent study found that intestinal tight junction protein disruption causes microbial translocation from the gut to the blood in ST-segment elevation myocardial infarction patients and mouse models. Intestinal bacteria were found in the blood of STEMI patients (*Lactobacillus*, *Bacteroides*, and *Streptococcus*) (Zhou et al., 2018). In a long-term study, Amar et al. (2013) discovered that the blood of patients with an acute cardiovascular event significantly decreased total bacterial DNA compared to a healthy cohort and increased taxa assigned to the *Proteobacteria* (Amar et al., 2013). 16S rRNA sequencing was used to characterize the microbial communities in the whole blood of CVD patients and healthy controls (barcoded ion sequencing). Despite no change in bacterial diversity at the phylum level, *Proteobacteria* increased while *Firmicutes* decreased. *Gammaproteobacteria*, *Bacillales*, and *Staphylococcaceae* saw significant increases at lower taxonomic levels, but *Pseudomonadaceae* was the only one to show a significant increase. The study also showed that bacterial DNA and circulating virome were abundant in CVD patients. There are significant variations at the species level in different studies as the subjects of the studies have different selection criteria. Furthermore, the different origins of sample processing (cellular blood DNA, RNA from whole blood, or DNA from blood plasma) and methodologies used to characterize blood microbiota (qPCR, pyrosequencing, amplicon, and metagenome sequencing) can affect the results of blood microbiota.

## Blood bacteria as potential biomarkers

Molecular technologies such as next-generation sequencing, metagenomics, and targeted detection methods have dispelled the myth that blood is a sterile environment. Circulating bacterial populations can be a considerable biomarker for diagnosing infectious and non-infectious diseases. Recently, several fascinating studies proved that the gut microbiome acts as a diagnostic biomarker for numerous diseases, including type 2 diabetes (Qiu et al., 2019), CVD, heart failure (Kazemian et al., 2020), liver cirrhosis (Qin et al., 2014), colorectal cancer, and pancreatic carcinoma (Veziant et al., 2021). Similarly, altered blood bacteria in type 2 diabetes and cardiovascular disease could serve as a potential biomarker. Sequencing methods can be helpful to reveal the association of circulating bacteria dysbiosis and liver diseases. Variations in blood bacteria have been observed, and therefore, changes in circulating bacteria at early stages of CVD (Dinakaran et al., 2014), atherosclerosis (Sato et al., 2014), type 2 diabetes (Amar et al., 2013), and other non-congenital diseases can be used as biomarkers. A separate study recommended that variation in blood bacteria could be used as a biomarker to diagnose nonalcoholic fatty liver diseases (Lelouvier et al., 2016) in obese individuals. Similarly, the concentration of 16S rDNA in the blood can be used as an early predictor of diabetes in a healthy population (Amar et al., 2011). Although the patient with type 2 diabetes mellitus has no obvious difference at phyla and class level from healthy individuals, the genera *Sediminibacterium* was abundant in diabetes patients while *Bacteroides* were higher in non-diabetic patients (Qiu et al., 2019; Anhê et al., 2020). Cardiovascular disease is associated with an increase in *Proteobacteria* phylum in blood, while an elevated *Actinobacteria : Proteobacteria* ratio is a characteristic of individuals with cardiovascular disease (Amar et al., 2013; Dinakaran et al., 2014).

Recently, circulating bacterial DNA was used to characterize rheumatoid arthritis (RA) and results showed that the taxonomic ecology of RA patients’ blood microbiota was different from ankylosing spondylitis (AS), psoriatic arthritis (PA), and healthy cases (Hammad et al., 2020). In Parkinson’s disease, Alzheimer’s disease, and type 2 diabetes mellitus patients, correlative light-electron microscopy has revealed an accumulation of LPS secreted by reactivated dormant blood bacteria in the blood, which may alter blood coagulation (Pretorius et al., 2016), systemic inflammation, and blood–brain barrier permeability, and affect the hematological system (Kell and Pretorius, 2018). Recently, a study related to the biomarkers of the human aging project explained that microbial blood DNA could be positively associated with the level of FFA (free fatty acids). FFA plays a crucial role in different immunological and physiological processes. Furthermore, blood bacterial DNA was positively correlated with increased leukocytes, blood insulin, and glucose (D’aquila et al., 2021). The remarkable agreement of data across separate investigations demonstrated the presence of a core blood bacterial population dominated by *Proteobacteria*, with *Actinobacteria*, *Firmicutes*, and *Bacteroidetes* present in a lower amount. Thus, changes in the microbial blood composition have been linked to pathological states, suggesting that the microbiota’s composition can serve as an early biomarker of disease risk.

## Concluding remarks and future perspectives

Years of ongoing research and advancements in molecular techniques enabled the study of unculturable bacteria from various settings such as soil, water, gut, and blood samples. All this has been done to refute the idea that blood is a sterile environment. Using these techniques, studies have reported the presence of blood bacteria in healthy and diseased conditions. However, at this stage, it is very confusing to state it as blood microbiota research has been marred by fundamental faults such as skin, reagent, and laboratory equipment contamination, casting doubt on the findings’ validity. Moreover, transient immune clearance and uptake of bacterial cells by dendritic or M cells are considered one reason for the presence of blood bacteria. Considering these entire issues, still, researchers use healthy human blood as negative controls to compare the dysbiosis of blood bacteria of diseased individuals. Despite the shortcomings of the studies, a consistent image of blood microbiota will emerge by adopting sophisticated advanced techniques, including microscopy and DNA and RNA sequencing analysis. As it turns out, the blood bacteria are dominated by *Proteobacteria*, with smaller proportions of *Actinobacteria*, *Firmicutes*, and *Bacteroidetes* present (Moriyama et al., 2008; Amar et al., 2013; Damgaard et al., 2015; Lelouvier et al., 2016; Païssé et al., 2016; Panaiotov et al., 2018). A healthy (non-diseased) human blood microbiota is contentious. The evidence presented here and the tendency toward comparing blood microbiota as analytical (positive and negative) controls in diseased and healthy individuals cannot rule out background contamination, immunological clearance of bacteria by blood cells, or temporary bacteremia events. The concept of a healthy (disease-free) human blood microbiota is relatively novel and disputed. This knowledge and the tendency toward comparing blood bacteria as analytical (positive and negative) controls in diseased and healthy individuals are insufficient to exclude the possibility of background contamination, immunological clearance of bacteria by blood cells, and temporary bacteremia episodes. Although some bacterial phyla are beneficial or non-pathogenic, they cannot be considered microbiota because they are not alive or capable of multiplying in blood. In reality, these bacterial populations can result from physiological health problems or the factors mentioned before.

More detailed and thorough studies will help to understand the role of blood bacteria dysbiosis in disease mechanisms and as a biomarker for the early diagnosis of different diseases. Circulating bacteria can also be used as a therapeutic tool to restore the altered blood bacteria and bacterial metabolites in different metabolic and infectious diseases. However, it is still crucial to assume that reported blood microbiota is a static or dynamic population of microbes as there are serious concerns to be addressed first, such as background contamination of reagents, phlebotomy, immune clearance, or temporary bacterial load due to transient bacteremia. We encourage future research that considers the addition of external bacteria with the “time” component. Geographic translocation, animal and insect bites, surgical histories, oral irrigation devices, and wounds can add additional bacteria to healthy human blood. All these factors transfer infectious and non-infectious bacteria to healthy humans. Circulating bacterial composition can be affected by age, geography, and socioeconomic level (e.g., access to nutritious food and healthcare services). What is most important is to design solid experimental designs that require minimum handling and avoid cross-contamination of skin, reagents, and equipment. Scientists must improve the sample collection, processing, and data production process. Microbial DNA from needles, vacutainers, chemicals, and other consumables can be analyzed to see if any possible contamination is present. Finally, blood microbiota research is essential for establishing bacteria and other microorganisms’ potential roles and functions in human blood. The accumulating data on hypothetical blood microorganisms in healthy individuals support the development of novel, therapeutically significant research avenues.

## Author contributions

MG and RL: Conceptualization, investigation, writing the original draft, and preparation. AM: Collection of data, reviewing, and editing. LG and HD: Writing the original draft and reviewing the manuscript. GW: Conceptualization, critical review and formatting of the final manuscript, and supervision. All authors contributed to the article and approved the submitted version.

## Funding

This study was supported by grants from 2020 Li Ka Shing Foundation Cross-Disciplinary Research Grant (2020LKSFG01E), the Natural Science Foundation of Guangdong Province (2021A1515012470), the Department of Education of Guangdong Province (2020KZDZX1083), and Shantou Science and Technology Bureau (Shanfuke[2020]88-STKJ2021197, Shanfuke[2020]53-51 and [2020]16-2).

## Conflict of interest

The authors declare that the research was conducted in the absence of any commercial or financial relationships that could be construed as a potential conflict of interest.

## Publisher’s note

All claims expressed in this article are solely those of the authors and do not necessarily represent those of their affiliated organizations, or those of the publisher, the editors and the reviewers. Any product that may be evaluated in this article, or claim that may be made by its manufacturer, is not guaranteed or endorsed by the publisher.
